# Supplementary material for: From sequence to enzyme mechanism using multi-label machine learning
Source: BMC Bioinformatics. 2014 May 19;15:150. doi: 10.1186/1471-2105-15-150 (PMC4229970; doi:10.1186/1471-2105-15-150)
Supplement: Additional file 2 — Java code of ml2db. Additional file ml2db_code.tar.gz contains the Java source code to run the multi-label machine learning experiments and save the results to database. The code’s Javadoc is included. [file 1471-2105-15-150-S2.zip › additional file 2/ml2db/ml2db/doc/main/ml2db/al/database/UtilityTable.html]

UtilityTable


JavaScript is disabled on your browser.


- Overview
- Package
- Class
- Use
- Tree
- Deprecated
- Index
- Help

- Prev Class
- Next Class

- Frames
- No Frames

- All Classes

- Summary:
- Nested |
- Field |
- Constr |
- Method

- Detail:
- Field |
- Constr |
- Method


main.ml2db.al.database

## Class UtilityTable

- java.lang.Object
- - uk.ac.standrews.utils.main.database.Table
  - - main.ml2db.al.database.UtilityTable

- ---

    

  ```
  public class UtilityTable
  extends uk.ac.standrews.utils.main.database.Table
  ```

  Class

  Version:
  :   8 Jul 2011

  Author:
  :   Luna De Ferrari ldf(at)st-andrews.ac.uk

- - ### Field Summary

    Fields

    | Modifier and Type | Field and Description |
    | --- | --- |
    | `static java.lang.String` | `UTILITY_FUNCTION` a string describing the utility metric used to pick the next instance to be labelled |
    | `static java.lang.String` | `UTILITY_TABLE_NAME` the name for the table to store the evaluation results |

    - ### Fields inherited from class uk.ac.standrews.utils.main.database.Table

      `m_writer`
  - ### Constructor Summary

    Constructors

    Constructor and Description

    `UtilityTable(uk.ac.standrews.utils.main.database.DbManager dbMan)`

    the rank by utility metric of this instance (rank is from best to worst)
  - ### Method Summary

    Methods

    | Modifier and Type | Method and Description |
    | --- | --- |
    | `uk.ac.standrews.utils.main.database.TableRow` | `createRow(int experimentId, int stepId, java.lang.String instance, double utilityMetric, uk.ac.standrews.utils.main.database.TableManager tableManager)` |
    | `uk.ac.standrews.utils.main.database.TableRow` | `createRow(java.lang.Object[] values, uk.ac.standrews.utils.main.database.TableManager manager)` |
    | `static java.util.Vector<uk.ac.standrews.utils.main.database.TableColumn>` | `getColumnDefinitions()` |

    - ### Methods inherited from class uk.ac.standrews.utils.main.database.Table

      `getColumnNames, getColumns, getDbManager, getTableCreator, getTableName, getTableReader, getTableRow, getTableWriter, hasKey, initialise, initialise, name, saveRow`
    - ### Methods inherited from class java.lang.Object

      `equals, getClass, hashCode, notify, notifyAll, toString, wait, wait, wait`

- - ### Field Detail


    - #### UTILITY\_FUNCTION

      ```
      public static final java.lang.String UTILITY_FUNCTION
      ```

      a string describing the utility metric used to pick the next instance to
      be labelled

      See Also:
      :   Constant Field Values


    - #### UTILITY\_TABLE\_NAME

      ```
      public static final java.lang.String UTILITY_TABLE_NAME
      ```

      the name for the table to store the evaluation results

      See Also:
      :   Constant Field Values
  - ### Constructor Detail


    - #### UtilityTable

      ```
      public UtilityTable(uk.ac.standrews.utils.main.database.DbManager dbMan)
      ```

      the rank by utility metric of this instance (rank is from best to worst)
  - ### Method Detail


    - #### createRow

      ```
      public uk.ac.standrews.utils.main.database.TableRow createRow(int experimentId,
                                                           int stepId,
                                                           java.lang.String instance,
                                                           double utilityMetric,
                                                           uk.ac.standrews.utils.main.database.TableManager tableManager)
      ```


    - #### createRow

      ```
      public uk.ac.standrews.utils.main.database.TableRow createRow(java.lang.Object[] values,
                                                           uk.ac.standrews.utils.main.database.TableManager manager)
      ```


    - #### getColumnDefinitions

      ```
      public static java.util.Vector<uk.ac.standrews.utils.main.database.TableColumn> getColumnDefinitions()
      ```


- Overview
- Package
- Class
- Use
- Tree
- Deprecated
- Index
- Help

- Prev Class
- Next Class

- Frames
- No Frames

- All Classes

- Summary:
- Nested |
- Field |
- Constr |
- Method

- Detail:
- Field |
- Constr |
- Method
